# Supplementary material for: Efficient population modification gene-drive rescue system in the malaria mosquito Anopheles stephensi
Source: Nat Commun. 2020 Nov 3;11:5553. doi: 10.1038/s41467-020-19426-0 (PMC7609566; doi:10.1038/s41467-020-19426-0)
Supplement: Supplementary file 3 — Reporting Summary [file 41467_2020_19426_MOESM3_ESM.pdf]

nature research

Corresponding author(s): Anthony A. James

Last updated by author(s): Oct 9, 2020

Reporting Summary

Nature Research wishes to improve the reproducibility of the work that we publish. This form provides structure for consistency and transparency in reporting. For further information on Nature Research policies, see our Editorial Policies and the Editorial Policy Checklist.

Statistics

For all statistical analyses, confirm that the following items are present in the figure legend, table legend, main text, or Methods section.

n/a	Confirmed	
☐	☒	The exact sample size (n) for each experimental group/condition, given as a discrete number and unit of measurement
☐	☒	A statement on whether measurements were taken from distinct samples or whether the same sample was measured repeatedly
☐	☒	The statistical test(s) used AND whether they are one- or two-sided<br>*Only common tests should be described solely by name; describe more complex techniques in the Methods section.*
☒	☐	A description of all covariates tested
☐	☒	A description of any assumptions or corrections, such as tests of normality and adjustment for multiple comparisons
☐	☒	A full description of the statistical parameters including central tendency (e.g. means) or other basic estimates (e.g. regression coefficient) AND variation (e.g. standard deviation) or associated estimates of uncertainty (e.g. confidence intervals)
☐	☒	For null hypothesis testing, the test statistic (e.g. F, t, r) with confidence intervals, effect sizes, degrees of freedom and P value noted<br>*Give P values as exact values whenever suitable.*
☐	☒	For Bayesian analysis, information on the choice of priors and Markov chain Monte Carlo settings
☒	☐	For hierarchical and complex designs, identification of the appropriate level for tests and full reporting of outcomes
☒	☐	Estimates of effect sizes (e.g. Cohen's d, Pearson's r), indicating how they were calculated

Our web collection on statistics for biologists contains articles on many of the points above.

Software and code

Policy information about availability of computer code

Data collection	No software used.
Data analysis	The following software packages were used:<br>CRISP-ID<br>GraphPad Prism 8.4.2<br>Trimmomatic v0.35<br>BWA-MEM v0.7.8<br>SAMtools v1.9<br>Picard Tools v1.96<br>PEAR v0.9.8<br>FASTX-ToolKit v0.0.14<br>MAFFT v7<br>Model fitting using Markov chain Monte Carlo (MCMC) methods<br>Mosquito Gene Drive Explorer (MGDrivE) model

For manuscripts utilizing custom algorithms or software that are central to the research but not yet described in published literature, software must be made available to editors and reviewers. We strongly encourage code deposition in a community repository (e.g. GitHub). See the Nature Research guidelines for submitting code & software for further information.

Data

Policy information about availability of data

All manuscripts must include a data availability statement. This statement should provide the following information, where applicable:

- Accession codes, unique identifiers, or web links for publicly available datasets
- A list of figures that have associated raw data
- A description of any restrictions on data availability

The full sequence of the plasmids used in this work is deposited in GenBank under the following accession numbers: MW030449 (pVG362_Aste-U6a-Swap3-gRNA), MW030450 (pVG363_Aste-U6a-Swap4-gRNA), and MW030448 (pVG344_Aste_kh2-MCRv3-Vasa-Cas9_SWAP).
Raw sequencing data is available in the Sequence Read Archive (SRA) database under BioProject PRJNA607757 and accession numbers SAMN14145944 (cage 1:3B, generation G0), SAMN14145945 (cage 1:3B, generation G8), and SAMN14145946 (cage 1:3B, generation G14).
A source data file is provided for raw data displayed in Supplementary Figures 1, 3 and 4 and Supplementary Table 3.
All other raw data are contained either within the manuscript main text and supplementary information.

Field-specific reporting

Please select the one below that is the best fit for your research. If you are not sure, read the appropriate sections before making your selection.

☒ Life sciences ☐ Behavioural & social sciences ☐ Ecological, evolutionary & environmental sciences

For a reference copy of the document with all sections, see nature.com/documents/nr-reporting-summary-flat.pdf

Life sciences study design

All studies must disclose on these points even when the disclosure is negative.

Sample size	Sample sizes were chosen to account for possible stochastic variation and provide statistical power. For example, we chose three cage replicated for each gene-drive release frequency to evaluate stochastic effects. The number of animals scored for phenotypes in the cage experiments, life-table and competition experiments were chosen to provide robust statistical analyses. All mosquitoes were counted to measure population sizes. All animals were chosen at random from each experiment except for those from the rare phenotype experiments. All mosquitoes with rare phenotype were analyzed.
Data exclusions	DNA sequencing data not meeting quality control defined in analytical software were excluded. No other data were excluded.
Replication	Replicates were performed in sufficient numbers (equal or more than three) to account for variation and provide statistical rigor. All replicates were successful inasmuch as they provided data and all were included in the analyses.
Randomization	All mosquito samples for propagating next generations in the cage trials were selected randomly except the first generation population cages with 1:9 release male mosquitoes. This design is based on our previous work (Pham et al., 2019) and they selected to reflect frequency of the gene-drive system. All subsequent selections in these cages were random. Randomization was not relevant to those experiments designed to look at specific phenotypes (for example, rare phenotypes).
Blinding	Blinding was not relevant to the studies because none of the data recorded were subjective. All scoring was either counting mosquito numbers or recording specific phenotypes. The phenotypes are unequivocal.

Reporting for specific materials, systems and methods

We require information from authors about some types of materials, experimental systems and methods used in many studies. Here, indicate whether each material, system or method listed is relevant to your study. If you are not sure if a list item applies to your research, read the appropriate section before selecting a response.

Materials & experimental systems

n/a	Involved in the study	
☒	☐	Antibodies
☒	☐	Eukaryotic cell lines
☒	☐	Palaeontology and archaeology
☐	☒	Animals and other organisms
☒	☐	Human research participants
☒	☐	Clinical data
☒	☐	Dual use research of concern

Methods

n/a	Involved in the study	
☒	☐	ChIP-seq
☒	☐	Flow cytometry
☒	☐	MRI-based neuroimaging

Animals and other organisms

Policy information about studies involving animals; ARRIVE guidelines recommended for reporting animal research

Laboratory animals	Anopheles stephensi Indian strain mosquitoes. This laboratory colony was originally obtained from M. Jacobs-Lorena (Johns Hopkins University) and has been maintained for >15 years at the University of California, Irvine (UCI). No vertebrate animals or animals requiring ethics review were used in these studies.
Wild animals	No wild animals were used in this study.
Field-collected samples	No field-collected animals were used in this study.
Ethics oversight	Our work with recombinant DNA and gene-drive organisms was reviewed and approved by the Institutional Biosafety Committees of the University of California, Irvine (UCI) and University of California, San Diego (UCSD). Approval for rearing mosquitoes exotic to the state was granted by the California Department of Public Health. In addition, insectary facilities and containment were reviewed, inspected and approved by the United State Department of Agriculture.

Note that full information on the approval of the study protocol must also be provided in the manuscript.
